# Injectable autologous chondrocyte transplantation yields sustained clinical mid‐term improvement in acetabular cartilage defects in femoroacetabular impingement: A comparative analysis from the German Cartilage Registry (KnorpelRegister DGOU)

**DOI:** 10.1002/jeo2.70971

**Published:** 2026-10-29

**Authors:** Moritz Riedl, Stefan Landgraeber, Steffen Thier, Wolfgang Zinser, Alexander Zimmerer, Ingo Jörg Banke, Wolfram Steens, Volker Alt, Stefan Fickert

**Affiliations:** ^1^ Department of Trauma Surgery University Regensburg Medical Center Regensburg Germany; ^2^ Department of Orthopaedics and Orthopaedic Surgery Saarland University Medical Center Homburg Germany; ^3^ Orthopädische Chirurgie Heidelberg ATOS Clinic Heidelberg Heidelberg Germany; ^4^ OrthoExpert Knittelfeld/Graz Austria; ^5^ Center for Orthopaedics, Trauma Surgery and Rehabilitation Medicine University Medicine Greifswald Greifswald Germany; ^6^ Clinic of Orthopaedics and Sports Orthopaedics, School of Medicine and Health TUM University Hospital, Technical University of Munich Munich Germany; ^7^ Department of Orthopaedics University Medicine Rostock Rostock Germany; ^8^ Orthopaedic‐Neurosurgery Center Recklinghausen Germany; ^9^ Sporthopaedicum Straubing Straubing Germany

**Keywords:** autologous chondrocyte transplantation, cartilage repair, femoroacetabular impingement, hip arthroscopy, matrix‐associated autologous chondrocyte transplantation

## Abstract

**Purpose:**

To evaluate mid‐term outcomes of injectable matrix‐associated autologous chondrocyte transplantation (MACT) for acetabular cartilage defects in femoroacetabular impingement (FAI), comparing two different ACT products (NOVOCART® Inject vs. Chondrosphere®) using data from the German Cartilage Registry.

**Methods:**

This retrospective analysis included 91 FAI patients (mean age 31.5 years) with isolated International Cartilage Repair Society (ICRS) Grade 3 or 4 acetabular cartilage defects treated arthroscopically with either NOVOCART® Inject (*n* = 48) or Chondrosphere® (*n* = 43), with a mean follow‐up of 41.8 ± 18.5 months. Clinical outcomes were assessed using the International Hip Outcome Tool (iHOT33) and Visual Analogue Scale (VAS) for pain at 6, 12, 24, 36 and 60 months. Clinically important outcome values (minimum clinically important difference, Patient Acceptable Symptom State, substantial clinical benefit) were calculated at 36 months. Subgroup analysis compared defect severity (ICRS Grade 3 vs. 4) and product‐specific outcomes.

**Results:**

Significant improvement in hip function (iHOT33) was observed across all follow‐up time points in both groups (*p* < 0.01), with no significant between‐group differences in iHOT33 scores or longitudinal trajectories. Within‐group VAS pain scores improved significantly over time in the Chondrosphere® group (*p* < 0.01), whereas the improvement in the NOVOCART® Inject group did not reach statistical significance; however, no significant difference in pain outcomes was found between the two products. At 12 months, patients with ICRS grade 3 defects had significantly higher iHOT33 scores (73.1 vs. 60.5) and lower VAS pain scores (2.1 vs. 2.8) than those with grade 4 lesions.

**Conclusion:**

Arthroscopic MACT was associated with clinical improvement in pain and function up to 5 years in FAI patients with acetabular cartilage defects, with no statistically significant differences detected between hydrogel‐based and spheroid‐based products; follow‐up data at later time points were more limited for NOVOCART® Inject. Although statistically significant pain improvement over time was observed only in the Chondrosphere® group, no significant differences in pain outcomes between the two products were detected.

**Level of Evidence:**

Level III.

**Abbreviations**
ACT: autologous chondrocyte transplantation
FAI: femoroacetabular impingement
ICRS: International Cartilage Repair Society
iHOT33: International Hip Outcome Tool 33
MACT: matrix‐associated autologous chondrocyte transplantation
MCID: minimum clinically important difference
PASS: Patient Acceptable Symptom State
SCB: substantial clinical benefit
VAS: Visual Analogue Scale

## INTRODUCTION

Chondral lesions of the hip joint are common in young, active patients and, given cartilage's limited intrinsic healing capacity, tend to progress if left untreated [11]. In cam‐type femoroacetabular impingement (FAI), repetitive abutment of the femoral head‐neck junction against the acetabular rim damages the acetabular cartilage and, without treatment, can lead to early osteoarthritic degeneration [8, 9]. As the presence and severity of acetabular cartilage defects strongly predict outcomes after hip preservation surgery [4, 13, 17, 24, 25, 30], effective cartilage management is critical to the long‐term success of FAI surgery [18].

Bone marrow stimulation techniques such as microfracture, although widely used in the knee, appear to yield inconsistent results in the hip [19, 21], and violation of the subchondral bone can compromise the osteochondral unit and impair the outcome of subsequent cartilage repair [2, 10, 14]. These findings have shifted interest toward hip‐specific techniques that preserve the subchondral plate.

Autologous chondrocyte implantation (ACT) has evolved through multiple generations and is now an established option for larger chondral defects in the hip [20, 22], with current German guidelines recommending cell‐based repair for focal lesions exceeding 2 cm^2^ [5]. Because hip arthroscopy offers limited visibility and working space, fixation techniques used in open procedures are impractical, and modern third‐ and fourth‐generation ACT products have therefore been designed to be self‐adhesive and deliverable through small cannulas without additional fixation [16, 26]. NOVOCART® Inject (TETEC AG) and Chondrosphere® (CO.DON GmbH) are two such injectable MACT products suitable for arthroscopic application in the hip; they rely on distinct biological principles—an in situ polymerizing hydrogel carrier versus self‐aggregating, scaffold‐free cartilage spheroids (see Materials and Methods section)—which could translate into different adhesion, maturation and clinical performance profiles. Early case series support the feasibility and short‐term efficacy of both approaches in the hip [6, 12, 15, 23], but it remains unclear whether these biological differences produce clinically meaningful differences in outcome.

To date, no direct comparative study of these two MACT products in the hip has been available, and higher‐volume, longer‐term data are needed to confirm durability of outcome. The German Cartilage Registry (KnorpelRegister DGOU) offers a real‐world dataset suited to this purpose [29]. This registry was therefore used to compare mid‐term clinical outcomes of arthroscopic MACT with NOVOCART® Inject versus Chondrosphere® in patients with FAI‐associated acetabular cartilage defects, hypothesizing that both products would yield significant improvement in patient‐reported function and pain, while exploring whether either technique offers a clinically relevant advantage over the other.

## MATERIALS AND METHODS

### Study design and patients

This study is a retrospective analysis of prospectively collected data from the hip module of the German Cartilage Registry (KnorpelRegister DGOU) of the German Society for Orthopaedics and Trauma Surgery (DGOU). The registry is a nationwide, multicenter observational database initiated in October 2014 to track outcomes of cartilage repair and joint‐preserving surgery in the hip, knee and ankle [29]. All participating centres obtained institutional review board approval, and patients provided informed consent for data collection. For the present study, all registry patients meeting the following inclusion criteria were identified: (1) age between 18 and 60 years; (2) diagnosis of cam‐type FAI with associated acetabular chondral damage; (3) a single (unifocal) full‐thickness cartilage defect in the weight‐bearing region of the acetabulum, graded 3 or 4 by the International Cartilage Repair Society (ICRS) classification; (4) treatment of the cartilage lesion with a matrix‐associated autologous chondrocyte transplantation (MACT) product and (5) minimum follow‐up of 12 months post‐surgery with available clinical outcome scores. Patients were excluded if they had any of the following: hip dysplasia (defined as lateral centre‐edge angle <25°) or other morphologic hip deformities apart from FAI; radiographic osteoarthritis (Kellgren–Lawrence grade >1) or substantial pre‐existing degenerative changes; bipolar ‘kissing’ lesions or multiple cartilage defects in the hip (only isolated acetabular lesions were considered); any full‐thickness chondral defect on the femoral head; avascular necrosis of the femoral head; or inflammatory or post‐traumatic arthritis. Patients undergoing concomitant labral or FAI‐correction procedures were not excluded and represent standard clinical practice in this cohort.

Data were contributed by 11 participating centres. Product choice varied by centre: three centres used both products, whereas the remaining eight centres used predominantly or exclusively a single product (three exclusively Chondrosphere®, five exclusively NOVOCART® Inject), reflecting local availability and surgeon preference rather than randomized allocation. Individual surgeon‐level case volume was not captured by the registry and could therefore not be analysed separately.

### Surgical technique

All patients underwent arthroscopic treatment of FAI combined with a two‐step autologous chondrocyte implantation procedure to address the cartilage defect. Hip arthroscopy was performed in the supine position under general anaesthesia and traction, using standard anterolateral and anterior portals to access the central and peripheral compartments. After diagnostic inspection, labral and chondral pathology were assessed. If an acetabular cartilage lesion of ICRS Grade 3 or 4 was confirmed and met the size criteria for ACT (>2 cm^2^), the procedure was initiated.

During the first stage, 2–3 cylindrical full‐depth cartilage biopsies were arthroscopically harvested from an uncompromised, non‐weight‐bearing area of the femoral head–neck. These cells were sent to the respective manufacturer's laboratory for processing. In patients treated with NOVOCART® Inject, the chondrocytes were isolated and expanded in monolayer culture for 3–4 weeks, then suspended in the proprietary albumin‐hyaluronan hydrogel matrix. For Chondrosphere® patients, the chondrocytes were expanded for 6–8 weeks, formed into spheroids by centrifugation and cultured in medium containing autologous patient serum as previously described, without an exogenous scaffold [22].

The FAI morphology and concomitant labral pathologies were treated either in the initial or the following intervention depending on the surgeon's preference. Cam deformities were addressed by arthroscopic femoral head–neck osteoplasty (offset correction), and pincer‐type deformities were treated with limited acetabular rim trimming (acetabuloplasty) as indicated. Labral tears were either refixed with suture anchors or debrided, depending on tear type and tissue quality. As this decision depended on the individual surgeon's approach, the exact distribution of staged versus same‐stage timing was not captured by the registry and could therefore not be quantified retrospectively.

The second‐stage surgery comprised the arthroscopic implantation of the cells. Under traction, the acetabular defect was debrided of any remaining unstable cartilage, creating stable vertical edges while preserving the subchondral bone plate. Loose flaps of the labrum or acetabular rim cartilage were also debrided.

For graft implantation, the joint was kept relatively dry by temporarily deflating the irrigation fluid, and the ACT product was delivered arthroscopically through the working cannula using a specialized application system, as previously described in detail [6, 12, 20, 23, 26]. Briefly, the NOVOCART® Inject hydrogel was injected to fill the defect flush with the surrounding cartilage and polymerized in situ within about one minute, whereas Chondrosphere® spheroids were applied dropwise and evenly distributed across the defect bed with a probe, achieving stable adherence within 10–20 min without additional fixation. Traction was released once the implant had set. Figure 1 illustrates both techniques.

**Figure 1:**
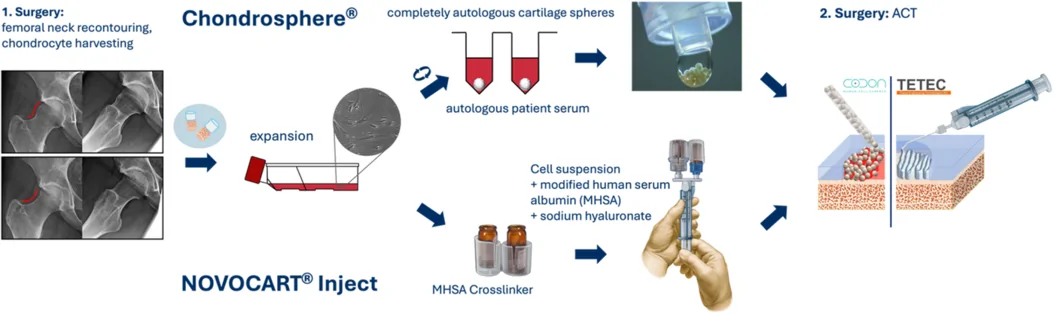
Illustration of the two‐stage ACT procedure in the hip joint using either NOVOCART® Inject (TETEC AG) or Chondrosphere® (CO.DON GmbH). ACT, autologous chondrocyte transplantation.

### Postoperative rehabilitation

The rehabilitation protocol was similar for both groups and followed standard guidelines for hip cartilage repair [20, 23, 26]. Patients were instructed to remain partial weight‐bearing (≈20 kg foot‐flat weight) on the operated limb for 6–8 weeks post‐surgery to protect the repair. Continuous passive motion (CPM) of the hip was utilized for 4–6 h per day during the first 4 weeks. Range of motion exercises were allowed as tolerated, avoiding impingement positions. If the labrum had been repaired, hip flexion was limited to 90° for the first 4–6 weeks to protect the repair. Stationary cycling without resistance was typically initiated at 2–4 weeks. Muscle strengthening and low‐impact activities (e.g., swimming) were gradually added after 8–12 weeks. Running and higher impact sports were deferred until around 6 months post‐op. All patients were kept on routine follow‐up schedules as per the registry protocol.

### Outcome measures

Clinical outcomes were assessed using patient‐reported outcome measures collected via the registry's electronic platform. The primary functional outcome was the International Hip Outcome Tool (iHOT33), a validated 33‐item questionnaire for young patients with hip pathology, scored from 0 (worst) to 100 (best hip function) [1]. The primary pain outcome was a Visual Analogue Scale (VAS) for hip pain (0 = no pain, 10 = worst pain imaginable), reflecting average pain during daily activities. To further evaluate specific symptoms, patient satisfaction and pain levels and to calculate the clinically important outcome values (CIOVs), supplementary questions detailed in Table 1 were incorporated into the questionnaire. Patients completed these questionnaires before the first‐stage arthroscopy (cartilage biopsy; preoperative baseline) and at subsequent intervals of 6, 12, 24, 36 and 60 months after the first‐stage arthroscopy. The interval between the first‐stage (biopsy) and second‐stage (implantation) procedures corresponded to the respective chondrocyte culture period (3–4 weeks for NOVOCART® Inject, 6–8 weeks for Chondrosphere®).

**Table 1:** Questions regarding patient satisfaction and pain for calculation of the CIOVs.

Question	Value
Are you satisfied with the outcome of the surgery?	1–5/not satisfied—very satisfied
How much did you benefit from the treatment?	1–5/impairing—very beneficial
How would you feel if you had to live with your current hip symptoms for the rest of your life?	1–5/not satisfied—very satisfied
Do you currently have pain in the operated hip?	1–4/no pain—strong pain
How often do you currently take pain medication?	1–5/never—daily

Abbreviation: CIOVs, clinically important outcome values.

### Statistical analysis

All data were analysed using SPSS Version 27 (IBM Corp.). Continuous variables were reported as means and SDs and categorical variables as counts or percentages. Differences in baseline data and score values between the different observation time points and treatment groups were assessed using paired *t* tests and one‐way analysis of variance (ANOVA) with a Bonferroni post hoc test on quantified data in case of normal distribution. If this condition did not apply, the Mann–Whitney *U* test and Kruskal–Wallis test were used. Chi‐squared test was used for the nominal demographic data. To account for repeated measurements over time and potential baseline imbalances between groups, a mixed‐effects model was applied in addition to pairwise comparisons. The mixed‐effects model included time, treatment group and the time‐by‐group interaction as fixed effects, with a random intercept per patient to account for repeated measurements; preoperative (baseline) values were included as the time = 0 measurement within this repeated‐measures structure. To address potential centre‐level confounding, a sensitivity analysis was performed adding centre as an additional random effect; the estimated between‐centre variance was negligible and did not materially change the treatment‐group or time‐by‐group interaction effects. Separately, multivariable linear regression was used to assess the association of defect size, age, BMI, sex, symptom duration, labral refixation and ICRS grade with absolute iHOT33 and VAS scores at each follow‐up time point. Statistical significance was defined as *p* < 0.05. CIOVs including minimum clinically important difference (MCID), Patient Acceptable Symptom State (PASS) and substantial clinical benefit (SCB) at 36‐month follow‐up were computed using distribution‐based and anchor‐based methods, respectively. The MCID was calculated as one‐half the standard deviation (SD) of the change in 36‐month iHOT‐33 scores. PASS and SCB values were determined through an anchor‐based method using receiver operating characteristic (ROC) analysis based on the questions in Table 1. MCID reflects a change‐score threshold (the pre‐ to 36‐month change in iHOT33), whereas PASS and SCB reflect absolute iHOT33 score thresholds at the 36‐month time point. The area under the curve (AUC) was calculated with a 95% confidence interval (CI). An AUC value greater than 0.8 and a 95% CI not including 0.5 are considered excellent characteristics for responsiveness. The Youden index was utilized to optimize sensitivity and specificity values and to determine the best cutoff values for SCB and PASS values. Missing follow‐up data were not imputed, and analyses were performed on an available‐case basis. Response rates at each time point are reported to ensure transparency regarding potential attrition bias. Given the observational registry design, loss to follow‐up is considered an inherent limitation and was accounted for by interpreting longitudinal trends with caution.

## RESULTS

At the time of data acquisition (January 2025), the German Cartilage Registry included 3376 patients in the hip section, with 137 patients treated with MACT. After exclusion of patients not meeting the inclusion criteria, 91 patients remained, of whom 43 patients underwent treatment with Chondrosphere® and 48 patients with NOVOCART® Inject. Figure 2 shows the described selection process of the current cohort in a flow chart. Figure 3 shows an intraoperative illustration of the two MACT techniques.

**Figure 2:**
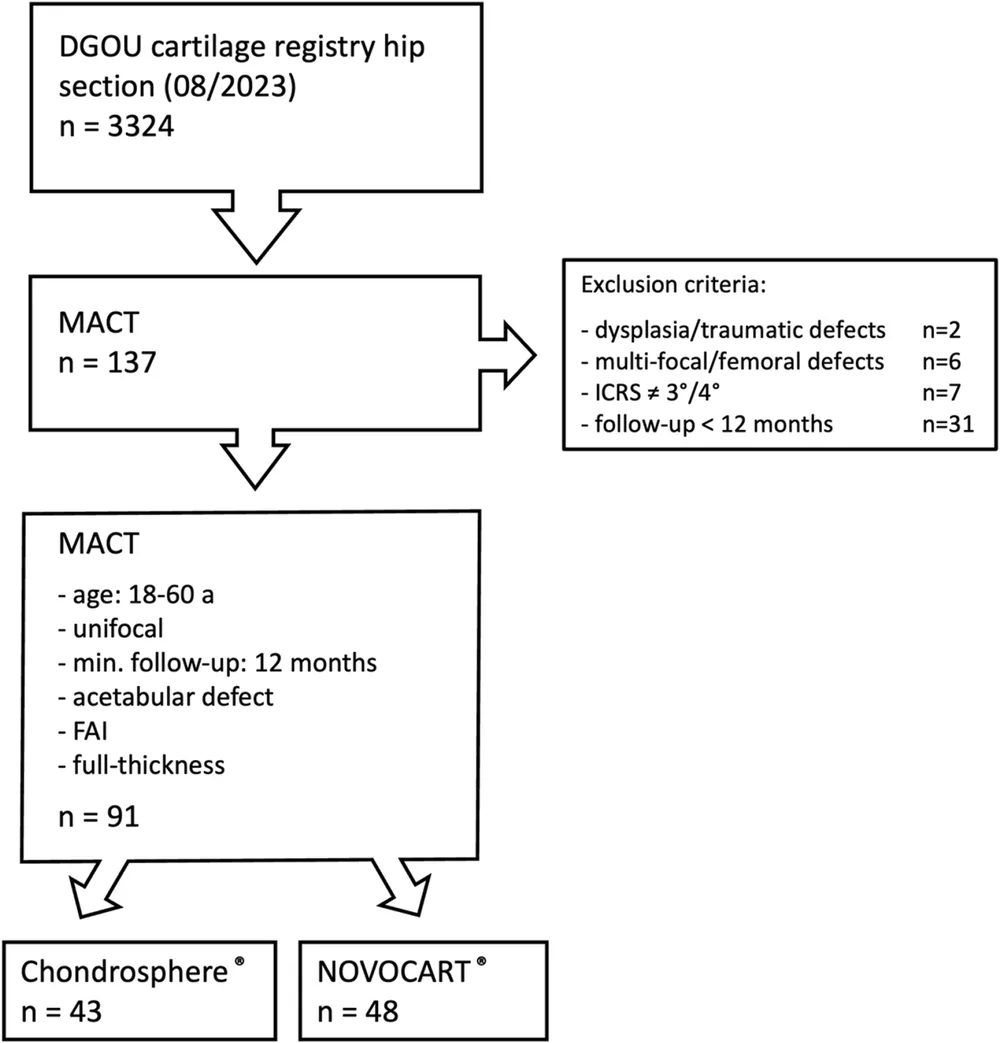
Flow chart of the inclusion process for the investigated patient cohort based on the data of the German Cartilage Registry (DGOU). FAI, femoroacetabular impingement; ICRS, International Cartilage Repair Society; MACT, matrix‐associated autologous chondrocyte transplantation.

**Figure 3:**
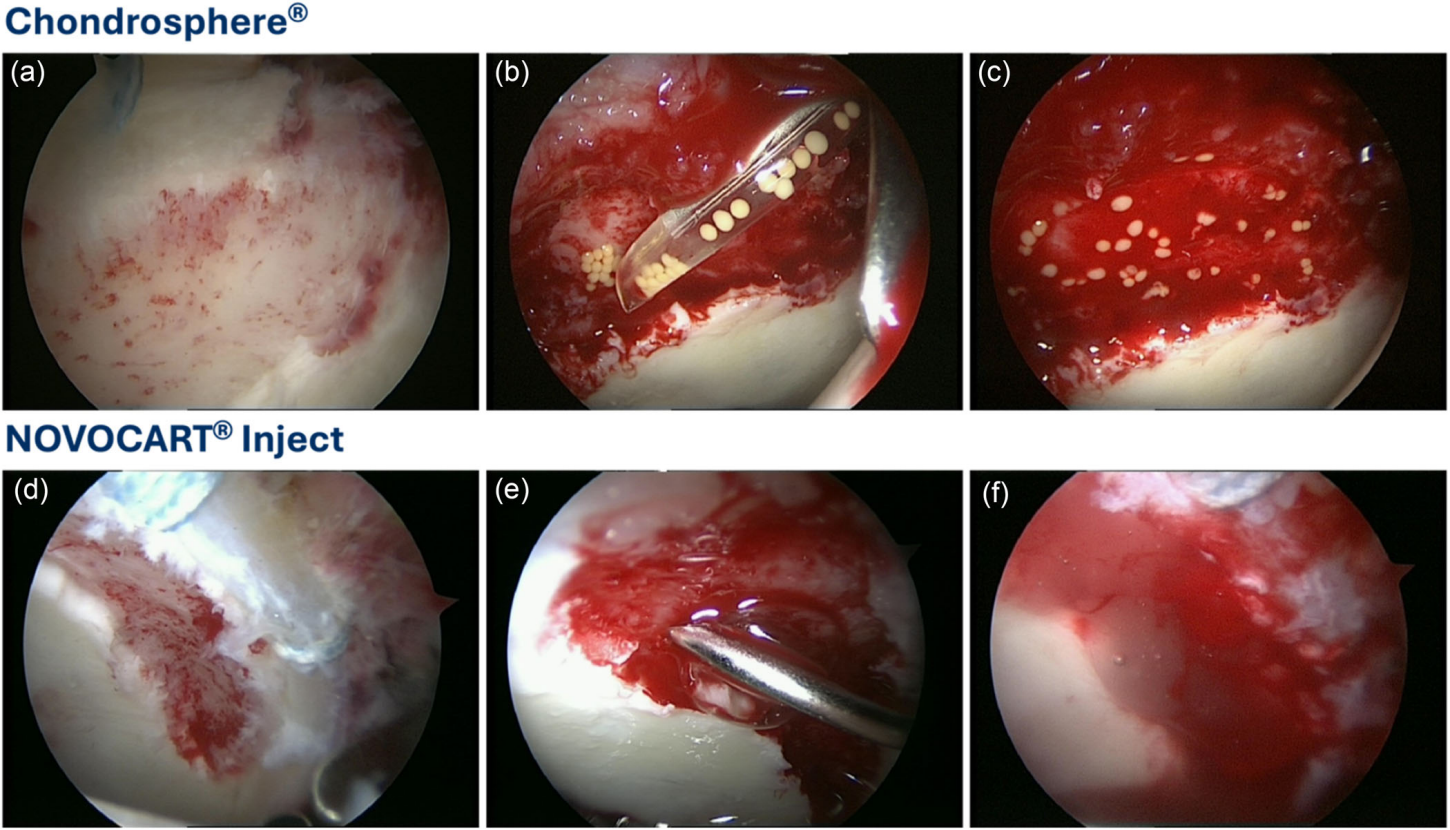
Intraoperative imaging of acetabular cartilage defects addressed either by Chondrosphere® (a–c) or NOVOCART® Inject (d–f). (a, d) Debrided cartilage defect prior to product application. (b, e) Application of the respective product. (c, f) Completed implantation with the product filling the defect.

The 91 included patients (12 women and 79 men) were aged 18–52 years (mean age 31.5 ± 7.7 years). Sex was included as a covariate in the regression analysis (see below) and was not significantly associated with clinical outcome; a separate sex‐stratified subgroup analysis was not performed given the small number of female patients (*n* = 12). All patients had a diagnosis of cam‐type FAI; 33 patients (36.3%) had a focal pincer component noted as well. By study design, all patients had a single focal acetabular cartilage lesion of ICRS Grade 3 (58.2%) or 4 (41.8%). The average chondral defect size was 3.4 ± 1.5 cm^2^. No patient had macroscopic damage to the femoral head cartilage. The mean follow‐up was 41.8 ± 18.5 months with a statistically significant difference in favour of the Chondrosphere® group (46.3 ± 19.5 months) compared to the NOVOCART® group (37.8 ± 16.6 months). Furthermore, there was a significantly higher rate of labral refixation in the NOVOCART® group (*p* = 0.001); otherwise, there were no significant differences between the treatment groups in terms of baseline characteristics. Both groups showed a similar distribution of sex, ICRS grade and intra‐articular pathologies (Table 2).

**Table 2:** Demographic data and baseline characteristics of the study population and the subgroups treated with NOVOCART® Inject and Chondrosphere®, including *p* values for intergroup comparison of the single characteristics.

		MACT	NOVOCART® Inject	Chondrosphere®	*p* value
		(*n* = 91)	(*n* = 48)	(*n* = 43)	
Sex					
Female	*n* (%)	12 (13.2)	9 (18.8)	3 (7.0)	n.s.
Male	*n* (%)	79 (86.8)	39 (81.3)	40 (93.0)	
Age (years)	Mean ± SD	31.5 ± 7.7	31.9 ± 7.0	31.1 ± 8.4	n.s.
BMI (kg/m^2^)	Mean ± SD	25.0 ± 3.2	24.9 ± 3.5	25.1 ± 3.0	n.s.
Smoking status					
Yes	*n* (%)	22 (24.2)	14 (29.2)	8 (18.6)	n.s.
No	*n* (%)	69 (75.8)	34 (70.8)	35 (81.4)	
Symptoms duration (months)	Mean ± SD	19.0 ± 12.5	20.5 ± 13.9	17.4 ± 10.9	n.s.
Follow‐up (months)	Mean ± SD	41.8 ± 18.5	37.8 ± 16.6	46.3 ± 19.5	0.029
Defect size (mm^2^)	Mean ± SD	341.8 ± 145.8	308.3 ± 130.7	370.6 ± 160.9	n.s.
ICRS grading					
Grade III	*n* (%)	53 (58.2)	29 (60.4)	24 (55.8)	n.s.
Grade IV	*n* (%)	38 (41.8)	19 (39.6)	19 (44.2)	
Underlying pathology					
cam deformity	*n* (%)	58 (63.7)	28 (58.3)	30 (69.8)	n.s.
Combined FAI	*n* (%)	33 (36.3)	20 (41.7)	13 (30.2)	
Labrum lesion					
Yes	*n* (%)	77 (86.3)	43 (89.6)	34 (79.1)	n.s.
No	*n* (%)	14 (13.7)	5 (10.4)	9 (20.9)	
Concomitant surgeries					
Labrum resection	*n* (%)	22 (24.2)	7 (14.6)	15 (34.9)	n.s.
Labrum refixation	*n* (%)	57 (62.6)	38 (79.2)	19 (44.2)	0.001
Acetabular trimming	*n* (%)	35 (38.5)	23 (47.9)	13 (30.2)	n.s.
Head–neck recontouring	*n* (%)	91 (100)	48 (100)	43 (100)	n.s.

Abbreviations: BMI, body mass index; FAI, femoroacetabular impingement; ICRS, International Cartilage Repair Society; MACT, matrix‐associated autologous chondrocyte transplantation; n.s., not significant; SD, standard deviation.

Preoperatively, the mean iHOT33 score for the entire cohort was 47.6 ± 19.2, indicating roughly 50% of full function on average, and the mean hip pain VAS was 4.0 ± 2.3 (moderate pain).

No statistically significant difference in baseline iHOT33 scores was observed between the treatment groups (Chondrosphere®: 44.5 ± 21.3 vs. NOVOCART®: 50.2 ± 16.7; *p* = 0.23). In contrast, baseline pain levels were significantly higher in the Chondrosphere® group (VAS: 4.4 ± 2.5) compared to the NOVOCART® group (VAS: 3.7 ± 2.1; *p* = 0.048). All patients underwent arthroscopic cam resection. Labral management (refixation vs. debridement) was decided intraoperatively based on tear morphology; exact numbers for labral and rim‐trimming procedures are given in Table 2.

The iHOT33 score in the overall cohort improved significantly from baseline at every follow‐up time point (*p* < 0.001; Figure 4). Both treatment groups improved significantly over time when analysed separately, with no statistically significant between‐group difference at any single time point (Figure 5).

**Figure 4:**
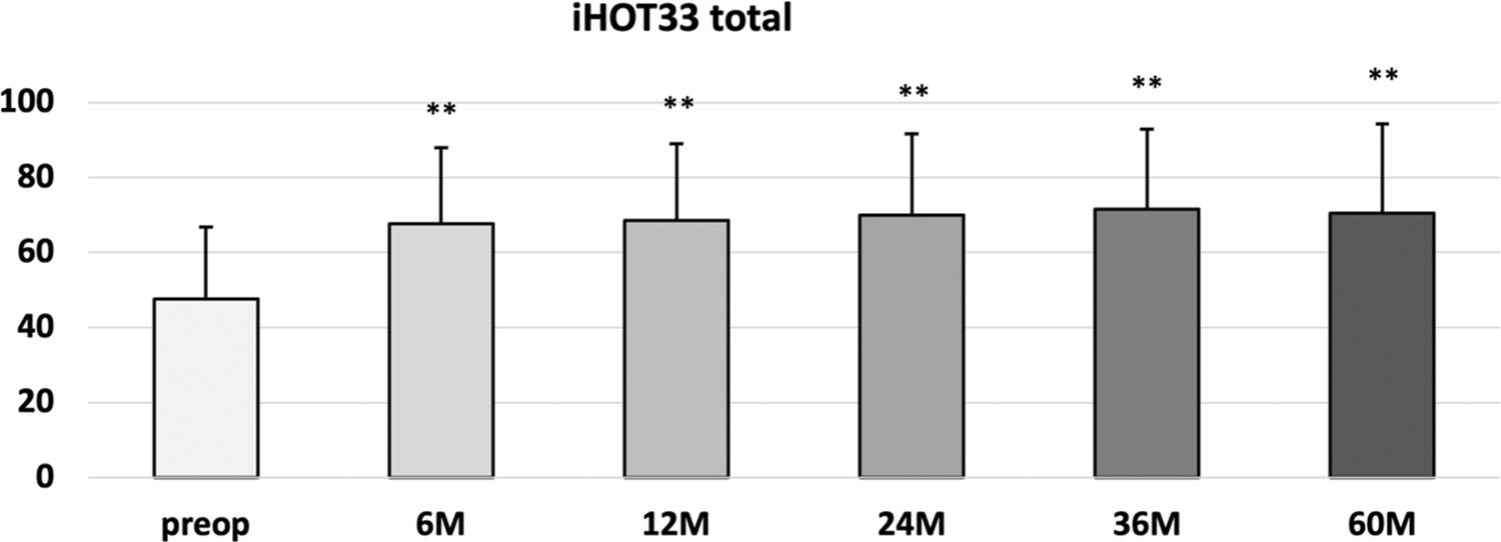
Outcome evaluation of the iHOT33 score in patients undergoing MACT of acetabular cartilage defects during hip arthroscopy from baseline to 60 months follow‐up. Whiskers indicate standard deviation. Asterisks mark statistically significant differences compared to preoperative scores (^⁎⁎^ *p* < 0.001). iHOT33, International Hip Outcome Tool 33; MACT, matrix‐associated autologous chondrocyte transplantation.

**Figure 5:**
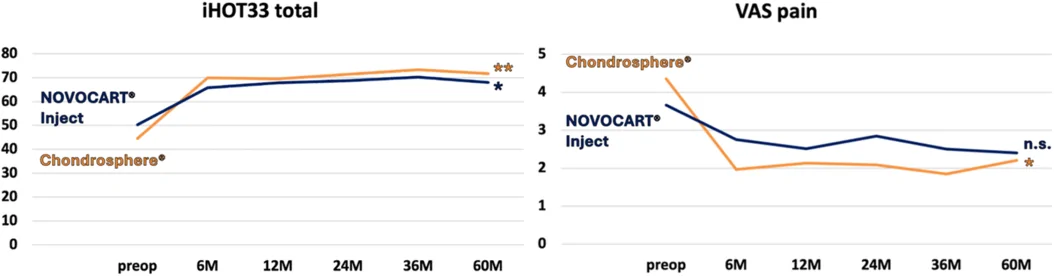
Development of pain and patient‐reported outcome over time in the two treatment groups (*p < 0.05; **p < 0.001, change to preop). iHOT33, International Hip Outcome Tool 33; n.s. not significant; VAS, Visual Analogue Scale.

Pain (VAS) likewise decreased significantly in the overall cohort at every follow‐up time point (Figure 6). By product, only the Chondrosphere® group showed a statistically significant pain reduction over time, while improvements in the NOVOCART® Inject group did not reach statistical significance at any time point (Figure 5). This should not be interpreted as clinical inferiority of NOVOCART® Inject, as point estimates likewise indicated pain reduction at every time point.

**Figure 6:**
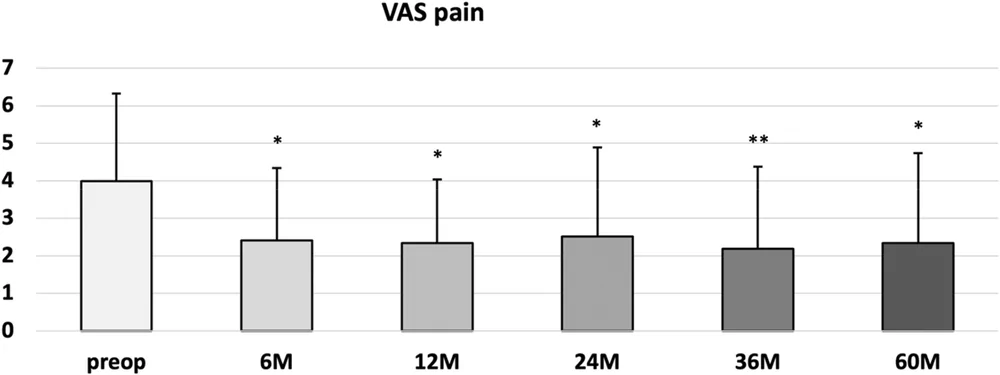
Results of the VAS for pain in patients undergoing MACT of acetabular cartilage defects during hip arthroscopy from baseline to 60 months follow‐up. Whiskers indicate standard deviation. Asterisks mark statistically significant differences compared to preoperative scores (^⁎^ *p* < 0.05; ^⁎⁎^ *p* < 0.001). MACT, matrix‐associated autologous chondrocyte transplantation; VAS, Visual Analogue Scale.

The mixed‐effects model confirmed a significant effect of time on both iHOT33 and pain outcomes (*p* < 0.001 for both). No significant main effect of treatment group was observed (*p* = 0.29, respectively, 0.15). Furthermore, no significant time‐by‐group interaction was detected (*p* = 0.41, respectively, 0.31), indicating comparable longitudinal outcome trajectories between NOVOCART® Inject and Chondrosphere®.

At the 36‐month follow‐up, CIOV analysis for the iHOT33 yielded an MCID of 10.6, a PASS of 55 and an SCB of 71.5, consistent with existing literature [27]. The majority of patients in both treatment groups exceeded these thresholds, indicating clinically meaningful improvement (NOVOCART® Inject: 66.7% MCID, 72.4% PASS, 51.7% SCB; Chondrosphere®: 90.5%, 83.3% and 62.5%, respectively; Figure 7). The mean 36‐month iHOT33 scores shown in Figure 7 did not differ significantly between groups (NOVOCART® Inject 70.1 ± 19.4 vs. Chondrosphere® 73.3 ± 24.0; *p* = 0.362), consistent with the absence of significant between‐group differences at other time points. As thresholds were derived from the overall cohort, subgroup findings should be interpreted descriptively.

**Figure 7:**
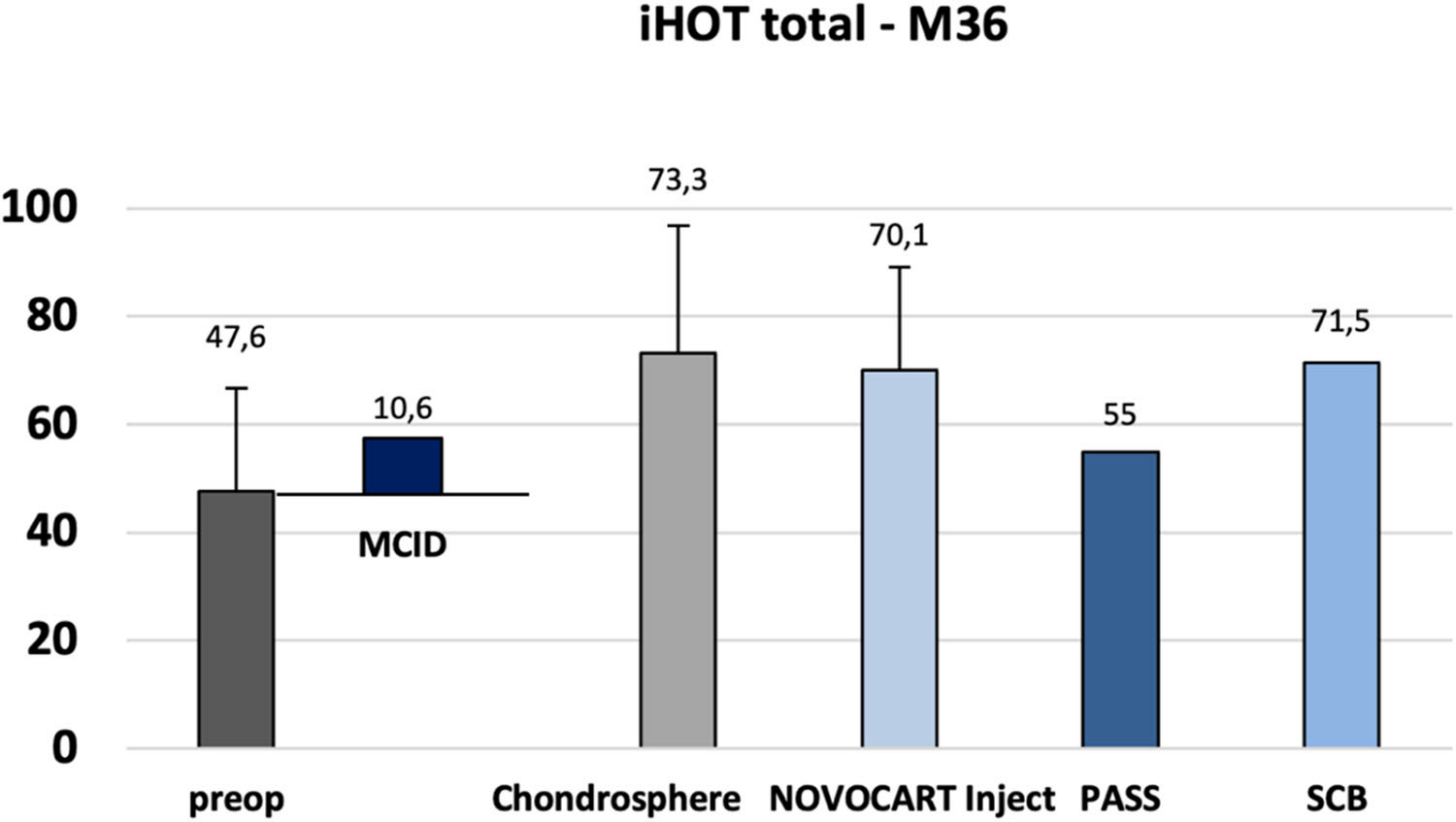
CIOVs of the iHOT33 36 months after hip arthroscopy with MACT (Chondrosphere® resp. NOVOCART® Inject) of the acetabular chondral lesion. Asterisks mark statistically significant differences (*p < 0.05). CIOV, clinically important outcome value; iHOT33, International Hip Outcome Tool 33; MACT, matrix‐associated autologous chondrocyte transplantation; MCID, minimum clinically important difference; PASS, Patient Acceptable Symptom State; SCB, substantial clinical benefit.

Regression analysis identified higher ICRS grade as associated with higher pain levels and lower iHOT33 scores at 6 and 12 months, but not at later follow‐up time points; defect grade was equally distributed between treatment groups. Change in iHOT33 and VAS was not significantly associated with defect size, age, BMI, sex or symptom duration. Although the rate of concomitant labral refixation differed significantly between treatment groups, it was not significantly associated with postoperative outcome scores.

To further investigate the effect of defect grade on clinical outcome after MACT in the hip joint, ICRS grade 3 and 4 defects were directly compared regarding pain level and iHOT33 outcome at each follow‐up time point (Figure 8). ICRS grade 3 defects exhibited statistically significantly higher iHOT33 scores and less pain than grade 4 defects 12 months postoperatively; differences at the remaining time points did not reach statistical significance. The mixed‐effects model demonstrated a significant effect of time (*p* < 0.001) and ICRS grade (*p* = 0.027) on clinical outcomes, as well as a significant interaction between time and ICRS grade (*p* = 0.042), indicating that the longitudinal outcome trajectories differed between ICRS grade 3 and grade 4 defects. This interaction suggests a more favourable iHOT33 trajectory for patients with ICRS grade 3 lesions over the observation period, consistent with the regression analysis identifying ICRS grade as a significant predictor of outcome at the earlier (6‐ and 12‐month) time points.

**Figure 8:**
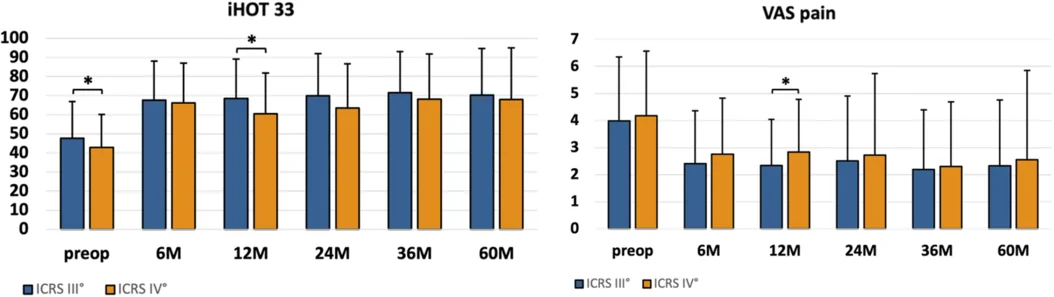
Comparison of ICRS grade 3 and 4 defects within the whole MACT cohort regarding the iHOT33 and VAS for pain. The bar chart shows a direct comparison between the defect grades on every follow‐up time point. Asterisks mark statistically significant differences (^⁎^ *p* < 0.05). ICRS, International Cartilage Repair Society; iHOT33, International Hip Outcome Tool 33; MACT, matrix‐associated autologous chondrocyte transplantation; VAS, Visual Analogue Scale.

Conversion to total hip arthroplasty occurred in 3 of 91 patients (3.3%) during the observation period (NOVOCART® Inject: 2/48, 4.2%, both converted at 24 months; Chondrosphere®: 1/43, 2.3%, converted at 60 months). Following conversion, no further patient‐reported outcome assessments were collected for these patients; consistent with the available‐case approach described above, their subsequent follow‐up time points were therefore treated as missing rather than imputed or carried forward, which may lead to a modest overestimation of outcomes at later time points, particularly for NOVOCART® Inject given its higher conversion rate. Revision arthroscopy was performed in 2 of 91 patients (2.2%), both in the Chondrosphere® group (2/43, 4.7%; NOVOCART® Inject: 0/48, 0%); the specific indications for revision could not be determined from the registry. Postoperative complications were not systematically captured by the registry at the individual patient level and could therefore not be reliably reported.

The adjusted response rates of iHOT33 at months 6, 12, 24, 36 and 60 were 76.9%, 76.9%, 61.5%, 58.2% and 47.3%, respectively; the corresponding numbers of patients with available data, overall and by treatment group, are summarized in Table 3. Figures 4, 5, 6, 7, 8 give a detailed overview of the iHOT33 and VAS results.

**Table 3:** Number of patients with available iHOT33 and VAS outcome data at each follow‐up time point, by treatment group.

Time point	NOVOCART® Inject (*n* = 48)	Chondrosphere® (*n* = 43)	Total (*n* = 91)
iHOT33			
6 months	39 (81.3%)	31 (72.1%)	70 (76.9%)
12 months	38 (79.2%)	32 (74.4%)	70 (76.9%)
24 months	32 (66.7%)	24 (55.8%)	56 (61.5%)
36 months	29 (60.4%)	24 (55.8%)	53 (58.2%)
60 months	15 (31.3%)	28 (65.1%)	43 (47.3%)
VAS			
6 months	40 (83.3%)	31 (72.1%)	71 (78.0%)
12 months	37 (77.1%)	31 (72.1%)	68 (74.7%)
24 months	31 (64.6%)	24 (55.8%)	55 (60.4%)
36 months	28 (58.3%)	25 (58.1%)	53 (58.2%)
60 months	15 (31.3%)	27 (62.8%)	42 (46.2%)

Abbreviations: iHOT33, International Hip Outcome Tool 33; VAS, Visual Analogue Scale.

## DISCUSSION

This study presents, to the authors' knowledge, one of the largest comparative series of injectable MACT in the hip, with follow‐up extending to 5 years. Both products led to significant and lasting improvements in patient‐reported outcomes, with an average iHOT33 increase of >20 points at 5 years (far exceeding the MCID), consistent with prior smaller studies [6, 12, 20, 23, 26]. These findings address the critical question of durability, confirming that the benefits of MACT were largely maintained at mid‐term follow‐up.

A key contribution of this study is the direct comparison of two MACT approaches—a hydrogel‐based MACT versus a scaffold‐free spheroid‐based MACT—in a relatively homogeneous patient population. In terms of hip function, no difference was found between NOVOCART® Inject and Chondrosphere®: Both groups improved significantly and to a similar extent on the iHOT33 at all evaluated time points. This suggests that, from a functional point, the tissue regeneration achieved by either method is comparably effective in restoring the joint's capacity for daily living and sport activities. These findings are in agreement with the short‐term results reported by Thier et al., who likewise saw no outcome difference between hydrogel‐ and spheroid‐based hip MACT at 19 months [26]. They are also congruent with registry data from the knee: Bumberger et al. recently compared Chondrosphere® versus NOVOCART® Inject in a matched cohort of knee defects and found equivalent overall clinical outcomes at 2 years, with both groups achieving favourable KOOS and IKDC scores [3].

In terms of pain relief, a divergence was observed: only the Chondrosphere® group achieved a statistically significant within‐group pain reduction over time, while the NOVOCART® Inject group did not, although both groups showed comparable pain improvement in absolute terms and no significant between‐group difference was found at any time point. This should be interpreted with caution, as it does not necessarily indicate that the hydrogel treatment is less effective, only that its average pain improvement was less pronounced in this cohort; the mixed‐effects model likewise supports comparable clinical outcomes between the two treatment modalities over time.

In essence, both groups achieved the primary goal of the surgery: to restore a pain‐reduced, well‐functioning hip joint that allows daily activities and sports with minimal limitations.

These between‐group comparisons should be interpreted with appropriate caution. Follow‐up duration differed significantly between groups (Chondrosphere®: 46.3 ± 19.5 months vs. NOVOCART® Inject: 37.8 ± 16.6 months; *p* = 0.029), and the proportion of patients with available data at the 60‐month time point also differed between groups, which may limit the comparability of long‐term outcomes. Baseline pain levels were higher in the Chondrosphere® group (VAS 4.4 ± 2.5 vs. 3.7 ± 2.1; *p* = 0.048), leaving more room for improvement and potentially contributing to the significant within‐group pain reduction observed only in this group. The rate of concomitant labral refixation was also significantly higher in the NOVOCART® Inject group (79.2% vs. 44.2%; *p* = 0.001); although regression analysis did not identify a significant association between labral treatment and postoperative outcome scores, a contribution of this imbalance to the divergent pain trajectories between products cannot be fully excluded.

In this cohort, a notable difference in clinical outcomes was observed when comparing patients with ICRS grade 3 and grade 4 acetabular cartilage lesions. Patients with grade 3 defects demonstrated significantly better postoperative improvement, particularly in pain reduction and functional outcome scores. Specifically, the VAS and iHOT33 scores at 12 months postoperatively showed statistically superior results in the grade 3 group compared to those with grade 4 lesions. The significant time‐by‐ICRS interaction in the mixed‐effects model suggests that defect severity not only influences absolute outcome levels but also affects the longitudinal recovery pattern following MACT. Patients with ICRS grade 3 defects demonstrated more favourable improvement trajectories, supporting the concept that earlier intervention, before further progression of the cartilage defect, may result in superior regenerative potential and clinical benefit. However, other factors associated with a greater overall disease burden in grade 4 defects may also contribute to this difference and cannot be excluded based on the present data.

These findings align with prior literature indicating that the extent of cartilage damage is a major predictor of treatment response. The reduced efficacy in grade 4 lesions may be attributed to a more compromised subchondral bone interface, reduced integration potential of the graft or an altered intra‐articular environment less conducive to cartilage regeneration. Moreover, full‐thickness defects may reflect a more advanced joint degeneration state, which could negatively influence the reparative process despite technically successful implantation.

Consequently, these results highlight the need for critical evaluation of defect severity when selecting candidates for MACT. If indicated, timely surgical treatment may be preferable to prolonged non‐operative therapy, although this cannot be established as a causal recommendation based on the present observational data. While injectable MACT remains a promising option in hip‐preserving surgery, patients with advanced cartilage damage (ICRS grade 4) should be counselled regarding the potentially limited clinical benefit. Studies are warranted to explore adjunctive strategies that may enhance outcomes in this challenging subgroup.

Beyond symptomatic improvement, a key theoretical advantage of MACT is the potential to restore more durable cartilage repair tissue while preserving the subchondral bone plate. This may be particularly relevant in young and active patients with FAI, in whom long‐term joint preservation rather than short‐term symptom control is the primary goal. However, although the present 5‐year data support sustained clinical benefit, they do not prove superior hyaline‐like cartilage regeneration or reduced osteoarthritis progression, which require structural and longer‐term outcome studies.

These results also highlight the importance of addressing the underlying bony impingement and preserving the joint's overall health. All patients underwent concurrent FAI correction; based on the underlying pathomechanism, this is considered an important component of the treatment strategy, as continued cam conflict could plausibly compromise the repair tissue, although this could not be directly tested in the present cohort since all patients received FAI correction. This combined approach—treat the cause (bone) and the effect (cartilage)—likely contributed to the success seen.

As a registry‐based retrospective study, there are several inherent limitations. First, treatment was not randomized; the assignment of NOVOCART® versus Chondrosphere® was likely influenced by centre preference or temporal availability. An attempt was made to account for baseline differences, and groups were found to be largely similar, but unrecognized selection biases could exist. Second, the patient‐reported outcomes, while standard and validated, introduce subjective elements. The iHOT33 and VAS, reported via an online system, were used; there could be response biases or drop‐outs. Furthermore, loss to follow‐up increased over time, with response rates declining to 47.3% at 60 months. This may introduce attrition bias, as patients with either particularly good or poor outcomes might be underrepresented at later time points. Third, the follow‐up duration of 5 years is moderate; it addresses mid‐term questions but not the crucial long‐term endpoint of preventing osteoarthritis. It is not yet known whether these repairs will hold up at 10–15 years or whether some will gradually deteriorate. Early data from knee ACT suggest over 50% graft survival at up to 20 years follow‐up [16], but whether the hip will mirror that remains to be seen. Continued surveillance of this cohort is needed to determine if and when the benefits start to wane. Fourth, a control group of, for example, natural history or debridement was not included in this analysis. This was beyond the scope of this study, and MACT superiority over other treatments cannot be directly claimed from this study alone. Non‐operative management (activity modification, physical therapy) remains the first‐line approach for symptomatic acetabular cartilage defects, while surgical alternatives include debridement, microfracture/bone marrow stimulation and autologous matrix‐induced chondrogenesis (AMIC). Comparative literature suggests MACT outperforms debridement at 5 years [7] and that MACT and AMIC yield better outcomes than microfracture in hips at mid‐term [28]. The timing of concomitant FAI correction and labral treatment was not fully standardized, as these procedures were performed either during the initial arthroscopy or at the second‐stage implantation according to surgeon preference. However, current evidence does not indicate a clear clinical advantage of either timing strategy, and this variability should therefore be interpreted as a potential but likely limited source of heterogeneity. Fifth, the registry did not systematically capture postoperative complications at the individual patient level; while conversion to total hip arthroplasty (3/91 patients) and revision arthroscopy (2/91 patients) were reliably recorded, the underlying indications for revision arthroscopy and the true overall complication rate in this cohort remain unknown. Sixth, the cohort was predominantly male (86.8%), reflecting the referral pattern to the participating centres, which may limit the generalizability of these findings to female patients.

## CONCLUSION

This study demonstrates that injectable MACT is a reliable and effective treatment for focal acetabular cartilage defects associated with FAI, with clinical improvement in hip function and pain observed up to 5 years postoperatively, although follow‐up data at later time points were more limited, particularly for NOVOCART® Inject. Both evaluated products, NOVOCART® Inject and Chondrosphere®, were associated with comparable improvement in function, and although a statistically significant within‐group pain reduction was observed only for Chondrosphere®, no significant between‐group differences were detected. Defect severity appeared to influence clinical success, with more favourable outcomes in ICRS grade 3 than grade 4 lesions. Overall, this study supports both hydrogel‐ and spheroid‐based MACT as viable options in hip‐preserving surgery, with careful patient selection and concurrent correction of bony impingement likely contributing to optimal outcomes, although this could not be directly tested in the present cohort.

## AUTHOR CONTRIBUTIONS

Moritz Riedl and Stefan Fickert contributed to study conception and design, statistical analysis, interpretation of the data and manuscript preparation. Stefan Landgraeber, Steffen Thier, Wolfgang Zinser, Alexander Zimmerer, Ingo Jörg Banke, Wolfram Steens and Stefan Fickert contributed to patient recruitment, data acquisition and critical manuscript revision. Volker Alt contributed to interpretation of the data and critical revision of the manuscript. All authors read and approved the final manuscript.

## FUNDING INFORMATION

The authors have no funding to report.

## CONFLICT OF INTEREST STATEMENT

The authors declare no conflicts of interest.

## ETHICS STATEMENT

The study was conducted in accordance with the Declaration of Helsinki and approved by the Institutional Review Board of the University of Freiburg (No. 520/14). Informed consent was obtained from all individual participants included in the study.

## Data Availability

The datasets generated and/or analysed during the current study are available from the corresponding author on reasonable request.
